# Timescape of disaster risk governance in contemporary Japan: Neither state of normalcy nor constancy in regulation

**DOI:** 10.1371/journal.pone.0215164

**Published:** 2019-04-18

**Authors:** Urbano Fra.Paleo

**Affiliations:** Research Institute for Sustainable Land Development (Interra), University of Extremadura, Caceres, Spain; University of South Carolina, UNITED STATES

## Abstract

Although the relationship between public policies and disaster risk is apparent, its nature is not so evident. The dominant model, the disaster management cycle, is based on the principle of response and return to normalcy. In addition, it is accepted that policies are based on constant legal development and that risk governance is responsive to successive disasters. The temporal pattern of large nature-triggered and technological disaster events in Japan since the end of WWII has been researched by measuring the duration of events and discontinuities between them as well as the development of the regulation of disaster risk. The evolutionary relationship between these two parameters and other political and economic factors was reconstructed through the notion of disaster timescape. Results do not support the notion of disaster cycle, nor a return to normalcy at the national scale, but a timescape of overlapping and successive events. Furthermore, there is no evidence of a clear association between major events and legal development on disaster risk, neither between this and economic or political crises. Nor is there continual evolution of regulation of disaster risk but, rather, a sequence of long periods of quiescence and acceleration more indicative of policy punctuation. The disaster timescape points to greater complexity with the interaction of multiple driving forces and an unstable balance that goes beyond a simple linear cause and effect. In the disaster timescape, there appear to be overlapping trajectories of environmental, social, political and economic processes.

## Introduction

The temporal dimension of disasters is linked to the debate on whether a disaster should be understood as an isolated event triggered by a natural or a technological process or whether it should, on the other hand, be regarded as a manifestation of the constant accumulation of vulnerability and exposure to hazard, as well as an issue of social distribution of risk and, therefore, of a structural nature. The debate between the incidental and the structural is not just disciplinary since it has relevant consequences in the design of public policies to reduce the risk of disasters [1].

Commonly, normalcy refers to the ordinary condition during which human activity is not interrupted by a disturbance, with special emphasis on major focusing events [2] and to the desired state, after shock, attainable by taking a series of pre-established steps. The disaster management cycle is the paradigm sustaining this approach, based on an ordered succession of response, recovery, mitigation and preparedness for disaster. Nevertheless, there is evidence that events overlap, the phases of the disaster vary greatly in duration, and sections of the same territory, economic sectors and social groups recover differently [3], which gives rise to heterogeneous disaster scenarios and a stratification of the affected communities [4]. In addition, disasters unequally activate mitigation and emergency preparedness. Hills [5] points that, rather than returning to normalcy, events are followed by unexpected crises that do not lead to the previous equilibrium, what Bos, Ullberg and 't Hart [4] called the “disaster after the disaster”. Neal [6] criticizes the cycle approach, the origin of which can be found in the work of Carr [7], after identifying the internal contradictions that emerge during recovery. Neal considers that a disaster is multidimensional and, rather than a sequence there is a continuum of phases [8]. According to Bankoff [9] the phases are not sequential, but may be running in parallel and, therefore, are indistinguishable. Moreover, the disaster management cycle does not take social time into consideration and is essentially deterministic. There is the time of the event, a social time [10], and a political time. The social time may even be different for different actors, with winners and losers throughout recovery [3]. Whereas the response to the event has a fairly defined temporal frame to save lives and satisfy the most basic needs following the event, recovery from disaster has an indeterminate time due to its complex, multidimensional and non-linear nature [11, 3]. First of all, it does not have a clear end point since the conditions prevailing before the event are not necessarily going to be recovered [11] given that lessons have been learnt, interests and priorities have changed and the socio-ecological system has shifted to a new state. Secondly, recovery is highly dependent on political will and agenda, and on available resources. Legal change will not come about if it does not receive political issue attention [12] required to place it on the political agenda in a dialectic between external pressure–through media and social mobilization- and an internal response to attract resources [13] to shape legal change. Likewise, it depends on the resilience of society, the community and of the individual, such that recovery is highly variable and may last decades [14, 15]. Chang [11] illustrates the broad range of recovery times in Kobe following the 1995 earthquake. A few weeks to restore the electricity supply, two for the telephone network, two and a half months for water and gas, six months for the railways, twenty-one months for the roads, six years for the retail sector, ten years to recover previous population levels, 75% of manufacturing production and 65% of construction, depending on the level of exports of the sector. Disparities have also been described by Smith and Birkland [15] between the poor area of Ninth Ward and the wealthier Lakeview in New Orleans in the recovery after Katrina.

However, time is usually interpreted and experienced differently. The co-existence of multiple processes associated with an event and ongoing environmental and social processes, all of different duration, with their multiple interactions, shape a disaster timescape. Adam [16] introduces the concept of timescape in order to take into account the social production of time, in addition to space. In this sense, Pollitt [17] considers that social sciences have treated the temporal dimension with indifference, failed to consider temporal features and placed particular emphasis on the study of time in management and public policies. However, Bankoff [9] observes that time was in fact considered a factor, particularly in the concept of disaster cycle.

Rubin, Renda-Tanali and Cumming [18] examine the relationship of disasters and policy outcomes in the United States, especially of major focusing events, as a continuous series of all kinds of events, both natural and technological, and of multiple associated policy outcomes (reports, laws, plans or strategies) in the form of timeline. In the same way, political reports [19, 20, 21] or academic reports [22] associate legal change with a certain disaster. The linear cause-effect approach seeks to visualize how the shortcomings of certain policies [13] are addressed after a disaster strikes. Events are usually represented as “episodic series of non-sequential critical moments largely unrelated to each other or to other forces at work in society” [9] and as triggers “that accelerate processes of change already underway in society, and where change itself is likely to precipitate further changes” [9], given that under certain conditions they bring about dramatic policy changes [2]. Nevertheless, the question remains as to whether a univocal association between major events and political changes can be made. Bankoff [9] argues that disasters are both sequential events and historical processes whose origin predates the appearance of the triggering factor, and that "the lineal trajectory of cause and effect […] becomes both temporally disrupted and physically dislocated.” [9]. Adam [23] applied this perspective to food risks and Ibrion et al [24] to seismic hazards. Timescapes and timelines are both concerned with the succession of temporal features, but the difference lies in that the former adopts the perspective of complexity.

Events are not a single but, to a greater or lesser degree, a multi-hazard process. For example, a typhoon entails intense wind and rainfall, floods, landslides and storm surge that, in turn, trigger other natural or technological hazards. The triple disaster in Japan in 2011 –or the Great East Japan Earthquake (GEJE)- triggered by an earthquake, which caused a tsunami and this, in turn, a nuclear meltdown, is a patent exemplar of a natech disaster [25].

Japan is located in an area exposed to multiple natural hazards, and its high level of economic development renders its society highly vulnerable to technological hazards. Since WWII, multiple events have sustained the representation of this vulnerability and has prompted political action through multiple and innovative disaster-risk reduction strategies. Nevertheless, the 1995 Great Hanshin-Awaji Earthquake (GHAE) and 2011 GEJE made it clear that, in spite of advancement, major events lead to substantial disruption, loss and damage.

The objective of this article is to examine the historical pattern of events in Japan since the Second World War and of disaster-risk regulation since WWII in order to compose the disaster timescape. First of all, an analysis is made of the temporal pattern of the major events according to their duration and time in-between. It goes on to examine the temporal pattern of disaster risk regulation and the key components of risk governance. Finally, a disaster timescape is outlined, based on the pattern formed by major events, policies, and economic, political and social processes.

## Methods and data

### The study of timescape

To reconstruct a timescape Adam [26] proposed a methodology that involved going beyond quantitative time, by examining the multidimensional context in which time and space are integrated and, accordingly, observing the phenomena that take place simultaneously and on different timescales. Following this approach, this study examines the intersection of multiple patterns and scales:

- The temporal distribution of the series of events with a higher number of fatalities (or casualties) caused by various hazards and the patterns of events and discontinuities,
- The spatiotemporal distribution of events in short time frames,
- The legal evolution, focusing on the key regulation instruments, of disaster-risk governance,
- The temporal pattern of key economic and political processes,
- The reconstruction of the timescape, by examining the intersections between the temporal patterns of major events and public policies.

This methodology recognizes “the temporal features of socio-environmental events and processes, charting temporal profiles in their political and economic contexts” and “the temporal complexity of socio-environmental existence” [26]. It is in line with the retrospective longitudinal analysis (RLA), proposed in the context of Forensic Investigations of Disasters (FORIN), in the sense that a retrospective longitudinal analysis of the pattern of disaster loss and damage is made, examining the “underlying organizational forms and institutions that condition choices and decisions“[27]. From among the six methodologies put forward by Pollitt [28], this approach seems to fit to what is known as organizational evolution perspective.

A disaster cannot be examined exclusively from a quantitative point of view; it must also be qualitative. Quarantelli [29] observes that in a disaster new forms of relationship among social organizations emerge and grow, whilst the organizations lose part of their autonomy their performance shifts, and closer cooperation between public and private institutions is induced. While recognizing this assumption, in this research the number de fatalities is an operational variable for interpreting the temporal pattern of major events and examining its relationship with the development of regulation in a long-term analysis, given that these disasters may influence public policies. This perspective is also based on the assumption that the set of conspicuous distanced crisis emergencies overlies a succession of more routinely closer minor emergencies which, as a whole, equal the impact of a large disaster. This is the case of the 4,117 fatalities in traffic accidents in 2015 [30]. Nevertheless, these lesser events are not examined in the study.

In order to identify the temporal pattern, the duration of events and of discontinuities or quiescences have been analyzed. Given that the duration of events has an exponential distribution and that of discontinuities is log-normal, the median was chosen as a descriptor and representative value, with the aim of discounting the weight of the outliers and the potential influence of time accuracy on the dating of events.

The series integrates both events with seasonality and, therefore, temporal regularity, and events that do not present a particular regular pattern, such as transport accidents -although some may be associated with seasonal adverse meteorological conditions- or industrial accidents, urban fires, intoxications, earthquakes and volcanic eruptions. Intense rainfall occurs twice a year, in Baiu, the season between early June and early July, and in the middle of September in the Shurin season, overlapping with the typhoon season from July to October. Heavy snowfall occurs around December and high temperatures run from June to September. Yet, the aim of the study is not to identify the degree of regularity of those events.

In order to examine the development of policies addressing disaster risk in Japan the main acts and amendments directly or indirectly affecting hazard management or a component of risk governance approved since 1946 were identified. Then, a subset was extracted of the key laws of a general nature relating either to the management of all disasters or to vulnerability, and of the sectorial laws focusing on each type of hazard, developed throughout the study period. They are 100 instances in the history of legal development. For the sake of methodological consistency norms approved before the war were excluded even though they are examples of early regulation, such as the Provision and Saving Act for Natural Disaster (1880), the River Act (1896), and the Erosion Control Act (1897) [31].

In order to measure the level of legal stability the total annual quiescence was calculated (*tq*_*t*_), estimating first the times of quiescence by intervals between successive enactments and/or amendments to each law (*tq*_*n*_), and then the time of total annual quiescence was calculated as the sum of the times of partial quiescences in each single year. *tq*_*t*_ in a certain year represents the sum of the value of quiescence in all the examined laws for that year.

*tq*_*t =*_ *tq_0 +_ tq_1 +_ tq_2 + …_ tq_n_*

To better understand the context of decision-making, and in addition to identifying key economic and political events, the evolution of the time in office of Prime Ministers since 1946 and of the inflation rate in Japan since 1956 were examined.

### Data

The main disasters in Japan between 1946 and 2016, that caused 10 or more fatalities, and were brought about by meteorological-hydrological events (typhoons, floods, landslides, heatwaves and intense snowfalls), volcanic events, earthquakes, urban fires, transport accidents and industrial accidents have been identified. The Emergency Events Database (EM-DAT) (Université Catholique de Louvain) [32] uses that threshold as one of the criteria to include a disaster in the database. Additionally, events of diseases caused by industrial pollution and by mass food poisoning in terms of casualties were included, as well as major nuclear accidents, even those without fatalities, due to the criticality of this type of events. The database records the type of event, its location, its date or time range and the number of fatalities or casualties, as well as the information source.

Data were collected from diverse sources, such as governmental documents, databases and scientific literature. They include the White Paper on Disaster Management in Japan [19, 20, 21], the Climate Change Monitoring Report from 2003 to 2016 [33], the Fire Prevention Administration Report [34], the Digital Typhoon Database [35], and the Annual Typhoon Reports [36].

The database was refined by cross-validating data with other sources of information, particularly EM-DAT (CRED 2018) and journalistic sources (The Japan Times and several international media), in order to secure the consistency of records. In total, 241 major events have been identified. The diverse sources present discrepancies, sometimes quite considerable, in the number of victims and in the dating of the event, particularly those of longer duration. In addition, it is not possible to distinguish victims caused by the natural or technological hazard in complex disasters such as typhoons, with victims directly caused by wind or intense rainfall, and indirectly by floods or landslides. Accordingly, in many cases it is difficult to differentiate between victims of the earthquake and of the tsunami in the flooded areas during the GEJE. The discrepancy, even between official sources, has multiple causes, among which are the link between official identification and qualification of the victims and the recognition of financial compensation, such as in the case of the victims of the Fukushima Daiichi Nuclear Power Plant accident [37], or even the delayed impacts. There are also important discrepancies in the chronology or time range of events among sources. Their statistical influence has been observed to be particularly important in the case of extended events, such as intense snowfalls and heatwaves which may last for weeks or even months, given that they affect the calculation of time discontinuities. In this sense, the study of slow-onset long-duration weather and climate extremes presents several challenges. In particular, the statistical influence of the process of recording events must not be underestimated [38] given that “the choices of the indicators and the setting of the thresholds are critical, as they condition the timeliness and effectiveness of the warnings” [39], and although heat-related deaths have been progressively clarified, disagreement remains regarding the number of fatalities reported [40]. In some cases, as the result of the application of different criteria, there may be sharp discrepancies among sources. For example, according to Nakano, Matsueda, and Sugi [41] the heatwave of 2010 caused 1,700 deaths, whereas EM-DAT counts 170. In these cases, the more conservative value was chosen.

To better deal with these uncertainties, instead of directly using the number of fatalities or casualties in the analysis, the logarithmic transformation allowed the attenuation, in comparative terms, of the discrepancies among sources, the comparison of the extreme values of small and major events by normalizing the distribution, and the coexistence of the number of fatalities and casualties in the same series.

The evolution of disaster risk regulation was first examined through 12 laws and then through grouping 40 laws or amendments in two comprehensive sets (disaster recovery and all-seismic). The prime ministers’ terms of office were taken from the webpage of the Office of the Prime Minister of Japan [42], and lastly, the historical Japanese annual inflation measured by the consumer price index (CPI) was taken from the database of Triami Media BV [43].

## Results and discussion

### Temporal and spatial pattern

**Fig 1:**
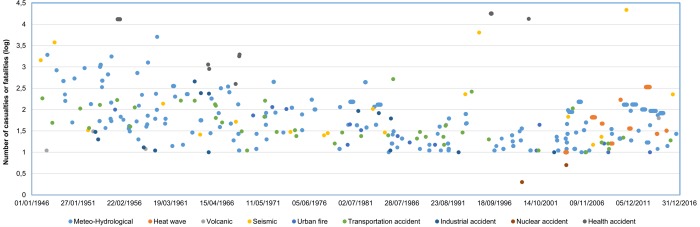
Flow of major disasters in Japan between 1946 and 2016. For better comprehension, a logarithmic transformation of the Y-axis values has been applied.

Beyond its proximate causes any disaster is distinctive due to the intervention of underlying processes and factors determining risk, the actors involved, the damage and losses caused and the course of disaster management. Nevertheless, disasters do not happen in isolation but, in a certain territory, occur at different spatial scales and locations. Singular major events in Japan between 1946 and 2016 developed as an undifferentiated flow of synchronic and diachronic events (Fig 1) over an even denser background of lesser events, while related and unrelated policy actions are taken, and social, environmental, economic and political processes happen. Thus, on a national scale events are not rare, but a component of the socioecological system that permanently determine policy action.

**Fig 2:**
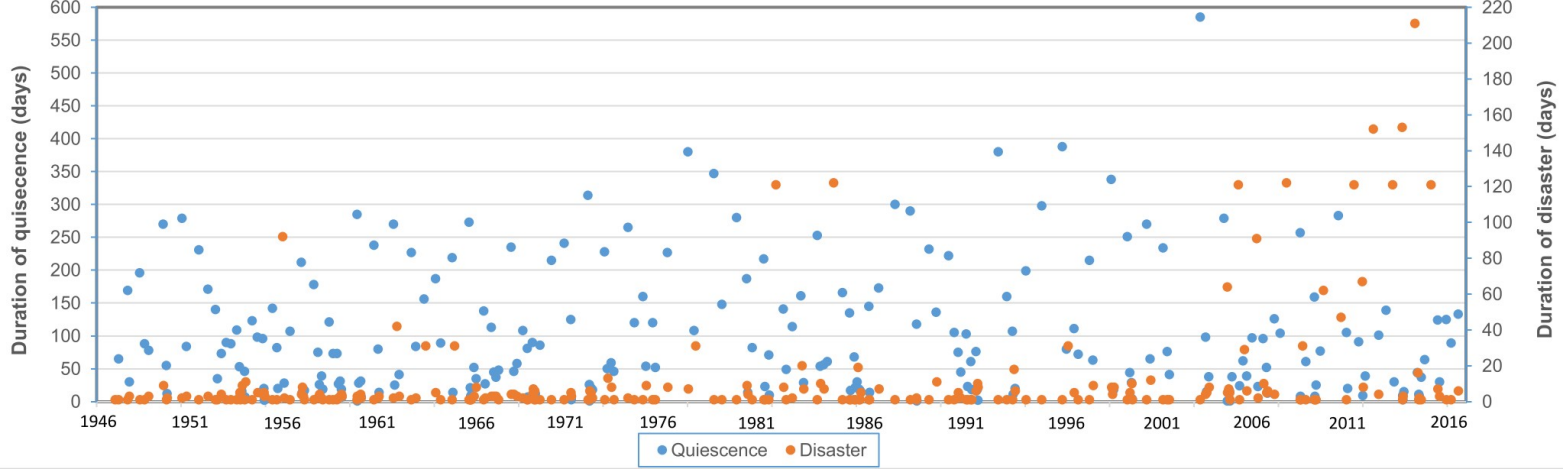
Duration of the periods of quiescence and of disaster events in Japan between 1946 and 2016.

Apparently, the flow of major events is continuous, but a more detailed analysis reveals that at this scale there are discontinuities of varying duration (Fig 2). On a national scale there are 2,731 days with events with a median duration of 2 days, alternating with discontinuities or quiescences whose median duration is 62.5 days. Given the brevity of quiescence, the response to disasters is virtually continuous on the national scale since, while response starts in the newly affected communities, recovery from past events continues elsewhere. There is no opportunity for a state of normalcy.

Some major episodes of the same class are close to each other, forming swarms of events that, having a long duration and a large geographical area coverage, increase the complexity of governance due to response and recovery taking place in various places, requiring and competing for resources. In 1966 there were four air accidents with many victims [44], two of them separated by just one day, and in 2004 five consecutive typhoons (numbers 15, 16, 18, 21 and 23) occurred within one or two weeks in between. Whereas the heat wave of July 2010 affected Southwestern Japan, and the intense snowfall of January 1963 in the central regions [21], are examples of combined large extent and long duration.

Four periods of quiescence of over a year’s duration stand out: between Typhoon 6 Rita (8/21-24/1975) and Typhoon 17 Fran (9/8-14/1976); between Great Hanshin-Awaji Earthquake (GHAE) (1/17/1995) and Toyohama road tunnel rockfall and collapse, Yoichi (2/10/1996); and the largest gap, between 2001 and 2003, without noteworthy events between the Myojo 56 building fire, Shinjuku, Tokyo (9/1/2001) and the explosion at the Nangoku Fireworks Co. in Kagoshima (4/11/2003).

Nevertheless, as the scale of analysis descends to events with less victims, quiescences become shorter and get closer to the duration of the events. Also, in recent years, events’ duration is becoming similar to quiescence’s due to the registration of long-duration events such as heatwaves and heavy snowfalls, which spread over weeks or months.

### Timescape and landscape

Disaster risk governance has multilevel temporal and spatial dimensions with various processes in different locations and on different spatial scales. Thus, although response, recovery and disaster preparedness take place on a local scale, risk reduction occurs at all scales since essential public policies are designed at the national level.

**Fig 3:**
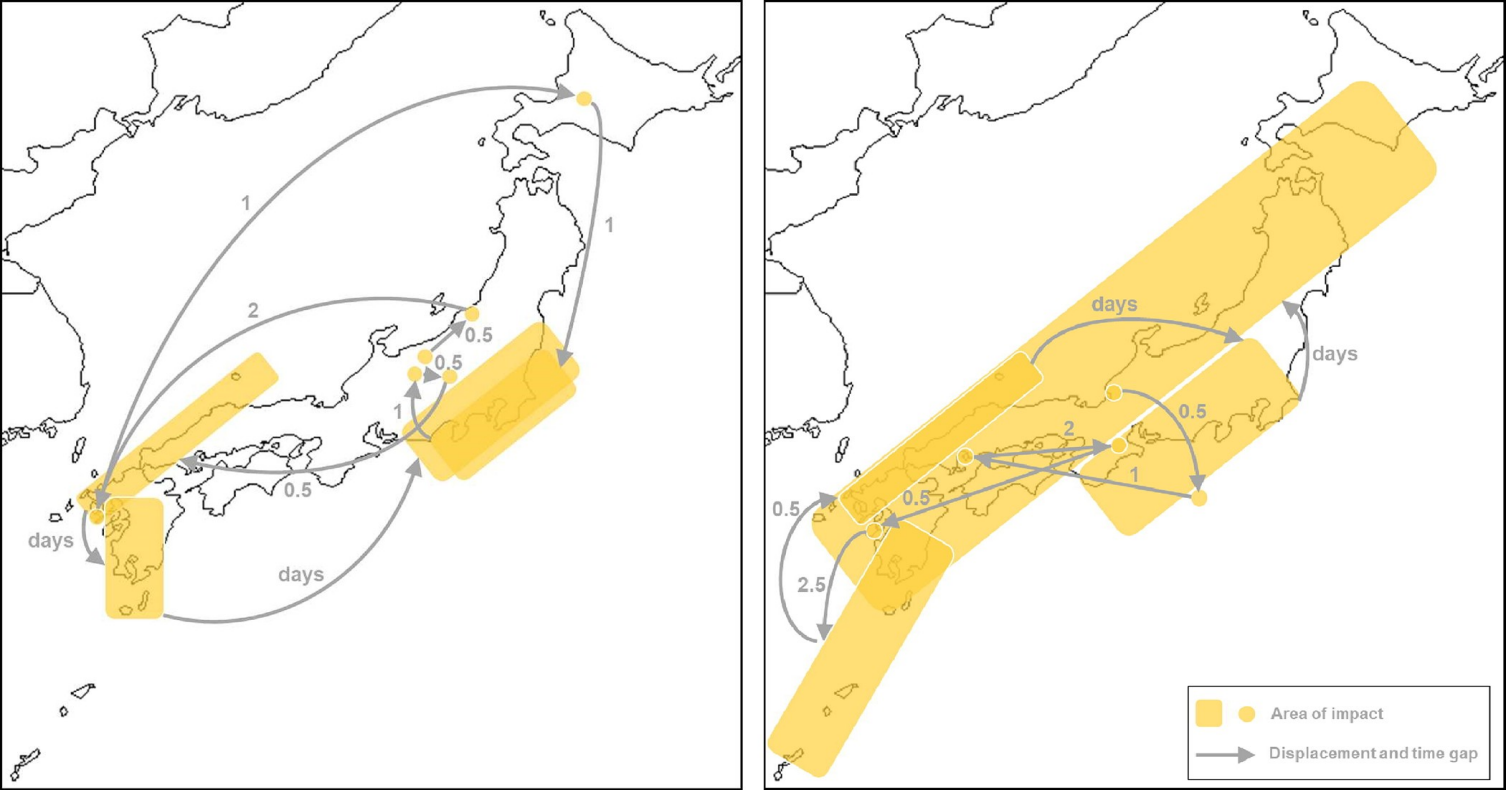
The timescape and landscape of disaster in Japan in 1985 (a) and 1991 (b) with the spatial displacement of major events, and the temporal gap (months) between each one.

The spatio-temporal pattern of major events in two years, 1985 and 1991 (Fig 3), illustrate the relationship between disaster timescape and landscape. The successive events have variable spatial coverage, are highly dependent on the triggering event, and their impact relocates throughout the country followed by processes of response and recovery. Relocation ranges from days to a few months and the sequence of disasters shapes a dynamic spatial landscape with multiple disaster timescapes, in which social groups, communities and economic sectors recover at different paces, that yield new social and spatial imbalances.

### Legal and policy change

Public policies dealing with disaster risk are multiple and heterogeneous because, ultimately, all policies influence people’s and various capitals’ exposure to hazard and their vulnerability. However, a core of regulations, those distinctively focusing on hazards or disaster management, is recognizable.

**Fig 4:**
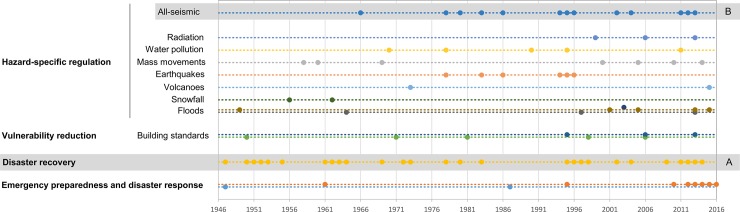
The flow of legal regulation of various hazards or components of disaster risk in Japan as the evolution of a single law or as a family of regulations (A and B).

Laws have a history that starts with their passing and is followed up with subsequent amendments to adapt them to the changing social, environmental and political conditions. Thus, some essential laws dealing with disaster risk governance in Japan and their revisions have been identified (Fig 4). In the sequence of enactments and amendments, distinct developmental patterns that have to do with early or late regulation, stability or mutability, long quiescence or swift change have been observed. Evolution ranges from a single change, such as the Act on Special Measures for Active Volcanoes, to seven changes in the case of the Basic Act on Disaster Control Measures. Thus, commonly, the legal framework is preferably sustained through refinement by amendments rather than by changing the legal paradigm.

After their enactment, some regulations go through long periods of stability before suddenly undergoing multiple changes in a very short time. For example, the 1961 Disaster Relief-Basic Action Disaster Control Measures Act was amended just once over a long time but after the GEJE and tsunami in 2011 it evolved very rapidly. The 1978 Large Scale Earthquake Countermeasures Act evolved quickly around 1995, coinciding with the GHAE, but then remained unchanged through a period of equilibrium or stasis. Notwithstanding, other laws regarding disaster recovery were passed in between, along with laws dealing with the seismic hazard in 2011. Likewise, legal stagnation does not mean adjustments were not being made through other policy instruments [18].

Another case is the regulation of the radiation hazard -Action Special Measures Concerning Nuclear Emergency Preparedness (No. 156 of 1999)- which was late in coming, taking into account that critical accidents had occurred both outside Japan, such as Three Mile Island (1979) and Chernobyl (1986), and in Japan (1991). In addition, although the regulation of nuclear energy production was introduced much earlier (Act No. 186 of 1955), only an Emergency Preparedness Guide (1980) was drawn up after the first of these events [22]. This upholds the statement of Nohrstedt [45] that “not all exogenous shocks translate into policy change”.

Integrating all of the norms and amendments that regulate disaster into two streams, a similar pattern can be seen. This is the case of the regulation of disaster recovery and all-seismic risk (A and B in Fig 4). In the case of the regulation of seismic risk a new instant of swift change occurs after 2011, and examining the evolution of the laws regulating recovery in various sectors of the economy and of the communities affected, another moment of rapid change came about at the beginning of the 1960s.

Despite there being occasional rapid changes, long discontinuities or periods of quiescence predominate. An example is the Act on Special Measures concerning Countermeasures for Heavy Snowfall Areas (No. 50 of 1962). This was an early regulation followed by a short evolution and then a long period of quiescence. This may reflect an efficient regulation of the management of this hazard, although it should be noted that it was completed by the more general regulation of disaster emergency preparedness and response. A similar pattern is seen in the regulation of flood and volcanic hazards. The quiescence of the Flood Control Act (enacted in 1949) is so long-lasting that it still remains in place even after close regulations, such as the River Act (already enacted in 1896, though amended in 1964), the Soil Conservation and Flood Control Urgent Measures Act (1960 *et sequentes*) and the Act on Countermeasures against Flood Damage of Specified Rivers Running Across Cities (2003).

A third group of regulations, on the reduction of vulnerability, has a slow and regular change pattern. This is the case of the Building Standards Act (1950), a law regulating the resistance of constructions. If the Act on Promotion of the Seismic Retrofit of Buildings (1995) is integrated into the sequence, no significant change is observed in the previous pattern. Water pollution regulation follows a similar pattern, shifting when the environmental problems deriving from the industrialization of Japan began to result in great epidemiological problems or pollution diseases [46] in the 1950s and 1960s.

**Fig 5:**
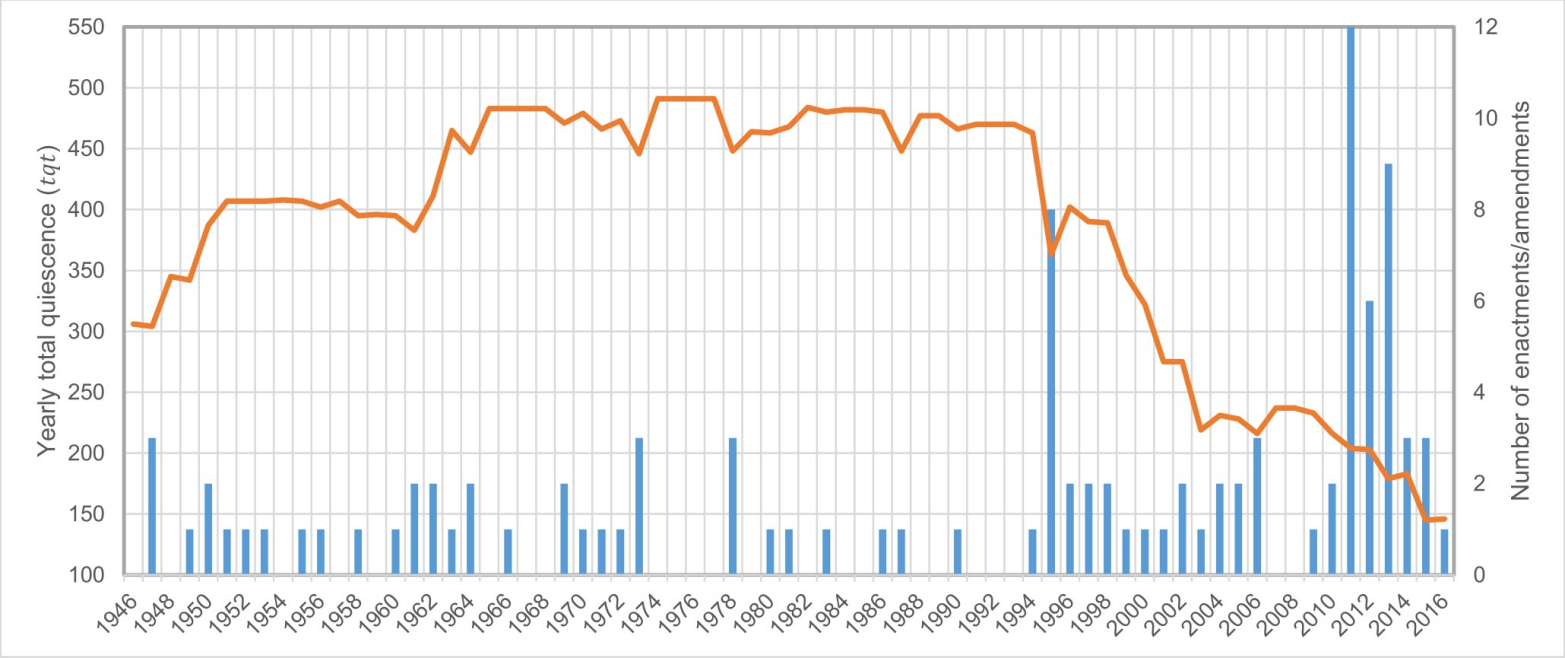
Evolution of disaster risk regulation in Japan after WWII in terms of the yearly total quiescence (*tq*_*t*_) (line) and the number of enactments/amendments per year (bars).

This flow of legal innovation, with enactments and amendments and periods of quiescence or legislative stability, shapes a political timescape parallel to the disaster timescape that, at some times, seems to respond to changes in the disaster timescape and at others is more resistant. The examination of the annual frequency of the enactment of laws and amendments regulating risk management as well as that of the total annual quiescence (*tq*_*t*_) (Fig 5) provides a more complete picture of the timescape of disaster risk policies in Japan since WWII and reveals three large evolutionary stages in the pattern of risk governance.

In the first phase, up to 1960, basic disaster relief and emergency preparedness policies began, to strengthen the resistance of structures and the management of hazards associated with meteorological processes such as floods, snowfalls and landslides. It was followed by a brief transition, a sudden change–the little acceleration- between 1960 and 1964, when a large number of laws were passed that were not modified for a long time. Between 1965 and 1995 there was a long period of continuity or stasis of the regulation previously approved, the great legal plateau, with the exception of the initial regulation of seismic, volcanic and water contamination hazards. This period of stability was followed -coinciding with the GHAE- by an acceleration in legal change, the great acceleration, with the enactment of numerous new laws and amendments, particularly in 1995 and 2011–2013, only briefly interrupted by a stagnation between 2007 and 2008. 2011 was an *annus horribilis* in terms of social, economic, political and even technological change [47], when a legal acceleration took place. The regulation of the nuclear radiation hazard began, and the aim of a swift recovery following the GEJE led to the enactment in two months of more than fifteen directly or indirectly related laws [48].

This time also saw significant international related developments. Three of the most influential global events on disaster risk reduction were sequentially convened in Japan in 1994, 2005 and 2015. However, since these conferences were organized within the framework of the United Nations, their respective outputs, the Yokohama Strategy and Plan of Action, the Hyogo Framework for Action and the Sendai Framework for Disaster Risk Reduction had, accordingly, a global scope. Their genesis was external [49], so that these events cannot be interpreted just in terms of Japanese national policy. The first World Conference on Disaster Risk Reduction stems from the International Decade for Natural Disaster Reduction (IDNDR) which, as follows from Lechat, was envisaged at the VIII International Congress of Earthquake Engineering, that was followed by a resolution of the UN General Assembly and by the appointment of a multidisciplinary group of experts from 24 countries to assist in preparing for the IDNDR.

This behavior is in line with the judgment of Walgrave and Varone [13] and Hathaway [50] who states that “legal change is characterized by periods of stability punctuated by periods of rapid change”, or policy punctuation [12]. According to Hathaway [50] change is delayed because the political system’s adaptative response is slower than the rate of change of environmental, social and economic systems.

Stability has been predominant in the evolution of disaster risk regulation in Japan, sustained by a continued regulation of social and economic recovery from disasters, only broken in two instances of swift change, the little and the great acceleration. The first stasis came after disaster response, vulnerability of structures and hazards associated with typhoons had been regulated, but without having yet regulated geological hazards, industrial pollution or nuclear radiation. This stability was broken soon after the impact of Typhoon 15 Isewan (1959) and a transition period began with a rapid regulation towards stability with few legislative changes. Stability was again interrupted in 1995 when a long transition towards a new state -whose nature is still unknown- began.

Reports such as the White Paper on Disaster Management in Japan [19, 20, 21] or HFA IRIDeS Review Report [22] adopt a cause-effect approach, associating every legislative change with a certain event. This is a suggestive idea but it poses the question of the point to which near or distant events as well as social, political and economic background processes have also affected each case. During periods of stability several large disasters did not have an appreciable impact on regulation, such as the Fukui Earthquake (1948, with 3,769 fatalities) and Typhoon Kanogawa (1958, with 1,269 fatalities) in the first stagnation; also Typhoon 10 Bess and the Nagasaki floods and landslides (1982, with 439 fatalities), and eight significant disasters in 1985 and 1991 with over 10 fatalities in the second stagnation. Thus, the number of victims does not always seem to be as much of a factor in determining policy action as does the criticality of the event or the type of hazard, since either catastrophic events like the GEJE, with over 21,000 fatalities, or the Tokaimura Plant (Tōkai) criticality accident, qualified as an INES (International Nuclear Event Scale) Level 4 in September 1999 with fewer than 10 fatalities, were followed by important changes in public policies. The former was followed by the enactment and amendment of a large number of laws, whereas the latter was followed by regulations such as the Act on Special Measures Concerning Nuclear Emergency Preparedness (1999) and the amendment of the Act on the Regulation of Nuclear Source Material, Nuclear Fuel Material and Reactors in 2000. At the same time, key social and political events arose during times of stability and, apparently, did not greatly affect the stasis, such as the contamination crisis between 1967 and 1969 [51], when victims began to litigate and claim compensation for the pollution diseases (Itai-Itai, Minamata, Niiagata Minamata and Yokkaichi COPD).

Nor can it be considered that disasters are the single driver of the acceleration of regulation. Walgrave and Varone [13] point out that one of the most important crises in the recent history of Belgium failed to induce political change, while a succeeding related event prompted major political change. In terms of the historical puzzle of Walgrave and Varone -much attention, little policy change versus little attention, major policy change- the GHAE favors the transition towards another state but it was not until the GEJE that it accelerated sufficiently to bring about a dramatic change in risk governance in Japan.

### Economic and political change

Beyond disaster events, some crises in Japan triggered by national or international developments might also have influenced the regulation of disaster risk. At the start of the great acceleration several key processes developed: in 1993 the *1955 political system* collapsed [52] with the loss of the parliamentary hegemony and majority of the Liberal Democratic Party of Japan since the end of the allied occupation, the crash of the bubble economy between 1991 and 1995 [53] and the sharp appreciation of the yen between 1994 and 1995 [54]. Soon afterwards commenced a period of flat nominal GDP in 1997 [53], the 1997 Asian financial crisis, the start of the Japan financial crisis in 1997–1998 [53], and the deflationary period (1998–2012) [53]. Then came the global financial crisis between 2008 and 2009, which led to the collapse of exports and the fall in industrial production.

**Fig 6:**
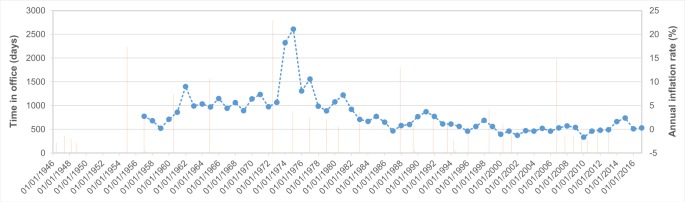
Evolution of the time in office of Prime Ministers (bars) and of the inflation rate in Japan (line) after WWII.

Just as a disaster is not always a trigger for regulation, neither can it be stated that an economic or political crisis necessarily lead to legislative acceleration. Calder [55] maintains that economic factors such as inflation and economic recession are necessary but are not sufficient to explain political crisis like those of 1949–54 or 1971–76. This author provides some clues as to causality when he points out that an intentional acceleration of policy response can take place following a crisis, as opposed to the resistance to change during intervals, since it is a circumstance that some governments take advantage of to create an economic state of emergency in order to favor the adoption of measures of neoliberal reform [56], given that “short-term interests can be important in explaining major policy change” [45]. Two processes may serve as an example of acceleration. The first is the peak of legislative innovation in 1961 during the Ikeda cabinet, once the crisis of the Security Treaty had concluded in 1960; and the second at the start of the 1970s following episodes of the pollution diseases. Nevertheless, the oil crisis in 1973, which was followed by a period of high inflation (Fig 6), was accompanied by a complete stagnation in the development of disaster risk regulation between 1974 and 1977 (Fig 5). The observation of Kilian [57] that the high inflation of the 1970s and beginning of the 1980s was not so much due to energy shocks, like the one in 1973, as to macroeconomic policies, given that the response of inflation in Japan to each energy crisis has been different, supports the observation of the difficulty in establishing immediate relationships of cause-effect, even within the economic subsystem.

There have been 27 prime ministers of Japan since 1946. If we take into account that the Prime Minister’s mandate is four years, there have only been four who have completed a mandate, discounting Shinzō Abe who was still in office in 2016. We identify four periods of policy stability, 1948–1954, 1957–1972, 1982–1987, and 2001–2006 (Fig 6), whereas the remaining political time was more turbulent with prime ministers who could not complete their mandate. Both times of political stability and instability coincided with intervals of legislative stability and in others with an acceleration of regulation.

## Conclusions

The time of disaster starts much before the event with the sustained prevalence of the drivers of vulnerability over the measures for the reduction of disaster risk (DRR) [58]. And it continues beyond it with the consequences in the middle and long term, policy action among them. Other disaster events, before and after, modify the risk conditions by causing losses and damage, so that the continuous accumulation and destruction of physical, environmental, social or human capital in turn change the conditions of future events. The disaster timescape is shaped by multiple layers of times, those of the event, environmental, social, economic and political times, which unfold in parallel and intersect with varying frequency, with sequences of quiescence and acceleration in a punctuated equilibrium [50]. Furthermore, the times of the event and of quiescence are variable, since they depend on the type of hazard and on its temporal (speed of onset, duration, frequency and temporal spacing) and spatial dimensions.

Kelman et al [59] question the assumption of the normal state to which society would return following disaster recovery, since society is changing continuously just as is the environment. The study shows that there is no evidence of a state of normalcy on a national scale, but rather Japan seems to be in a constant state of unrest, an unstable balance, with successive events or events occurring in parallel, which does not support a cyclic disaster management pattern.

Nor is there evidence of constancy in the legal evolution of disaster management. Quite the contrary, an alternation between periods of stagnation and of acceleration in the development of regulation is observed. This pattern matches a punctuated equilibrium [50] in public policy. In general, there was a dominance of stability broken at the start of the 1960s when a small regulatory acceleration took place and in 1995 when there was a long transition towards a new equilibrium in risk governance still in definition.

Factors and events that shape stability and acceleration are multiple and contingent, sometimes apparently unrelated, but with complex interactions. According to Becker [60] “disaster risk is a complex issue not only because it includes factors from all spheres of society, but also because many of these factors are interdependent on each other”. To associate a political change with a certain recent disaster adopting a linear cause-effect approach is suggestive, but is a simple interpretation of a reality where a heterogeneous set of driving forces, among them the incessant flow of events, proximate causes and the last event, determine the development of policies. An interesting area for future research concerns the examination of the political process and its interpretation made by those political actors who directly intervened in legal change.

This temporal pattern, or disaster timescape, does not support the view of a disaster management cycle, which examines disasters through a reductionist lens, in isolation, as if a certain disaster had nothing to do with other disasters. On the contrary, a temporal direction can be identified [16] since social groups and territories follow dissimilar recovery trajectories [3], in such a way that past events, though distant in time and with their effects attenuated, influence the present and the future. The disaster timescape also has a spatial dimension, with changes in the landscape brought about by disasters that engender territorial, social and economic imbalances. Similarly, there is no single time, but rather multiple times that interfere, extend and shorten. Adam [16] illustrates this with the decoupling between industrial time and the time of the pollution diseases, because of the latency between the pollution emission and disease cases. In addition to other latencies, such as the scientific time passed between causes and effects begin to be investigated and results are obtained; the judicial time since the lawsuits are filed and cases are sentenced; or the political time that passes before industrial pollution is regulated.

Contrary to what the paradigm of the disaster management cycle states, i.e. a stable balance occasionally broken by an event that is restored by human intervention, there is evidence of an unstable balance on a national scale in the disaster timescape of Japan since the end of the Second World War.

## References

[1] Fra.Paleo U. Introduction In: Fra.Paleo U, editor. Risk governance The articulation of hazard politics and ecology. Dordrecht: Springer; 2015 pp. 1–16.

[2] Birkland T. Focusing events mobilization and agenda setting. Journal of Public Policy. 1998; 18:53–74.

[3] Tierney K, Oliver-Smith A. Social dimensions of disaster recovery. Int J Mass Emerg Disasters. 2012; 30(2):123–146.

[4] Bos UK C, Ullberg S, 't Hart P. The long shadow of disaster: Memory and politics in Holland and Sweden. International Journal of Mass Emergencies and Disasters. 2005; 23(1):5–26.

[5] Hills A. Seduced by recovery: The consequences of misunderstanding disaster. Journal of Contingencies and Crisis Management. 1998; 6:162–70.

[6] Neal DM. Reconsidering the phases of disaster. International Journal of Mass Emergencies and Disasters. 1997; 15(2):239–264.

[7] Carr LJ. Disaster and the sequence-pattern concept of social change. The American Journal of Sociology. 1932; 38(3):207–218.

[8] de Ville de Goyet C. Information gaps in relief recovery and reconstruction in the aftermath of natural disasters In: Amin S, Goldstein M, editors. Data against natural disasters: Establishing effective systems for relief recovery and reconstruction. Washington DC: World Bank 2008; pp. 23–58.

[9] Bankoff G. Time is of the essence: Disasters vulnerability and history. International Journal of Mass Emergencies and Disasters. 2004; 22(3):23–42.

[10] Neal DM. Social time and disaster. Int J Mass Emerg Disaster. 2013; 31(2):247–269.

[11] Chang SE. Urban disaster recovery: a measurement framework and its application to the 1995 Kobe earthquake. Disasters. 2010; 34(2):303–327. 10.1111/j.1467-7717.2009.01130.x 19863570

[12] Baumgartner FR, Jones BD. Agendas and instability in American politics. Chicago: University of Chicago Press; 1993.

[13] Walgrave S, Varone F. Punctuated equilibrium and agenda-setting: Bringing parties back in policy change after the Dutroux Crisis in Belgium. Governance. 2008; 21(3):365–395.

[14] Sword-Daniels VL, Twigg J, Loughlin SC. Time for change? Applying an inductive timeline tool for a retrospective study of disaster recovery in Montserrat West Indies. International Journal of Disaster Risk Reduction. 2015; 12:125–133.

[15] Smith G, Birkland T. Building a theory of recovery: institutional dimensions. Int J Mass Emerg Disasters. 2012; 30(2):147–170.

[16] Adam B. Timescapes of modernity: The environment and invisible hazards. London: Routledge 1998.

[17] Pollitt C. Time, policy, management: Governing with the past. Oxford: OUP 2008.

[18] Rubin CB, Renda-Tanali I, Cumming WR. Disaster time line: Major focusing events and US outcomes (1978–2006). TR News 250; 2007 http://www.trb.org/Publications/Blurbs/158982.aspx

[19] Cabinet Office Japan. White Paper on Disaster Management in Japan 2015. Government of Japan 2016.

[20] Cabinet Office Japan. White Paper on Disaster Management in Japan 2016. Government of Japan 2017.

[21] Cabinet Office Japan. White Paper on Disaster Management in Japan 2017. Government of Japan 2018.

[22] IRIDeS. HFA IRIDeS Review Report Focusing on 2011 Great East Japan Earthquake. Tohoku: Tohoku University 2014.

[23] Adam B. Industrial food for thought: Timescapes of risk. Environmental Values. 1999; 8(2):219–238.

[24] Ibrion M, Mokhtari M, Parsizadeh F, Nadim F. Timescape of the earthquake disasters in Iran: The intricacies of earthquake time and earthquake disaster risk reduction. Geografiska Annaler: Series A Physical Geography. 2015; 97:197–216.

[25] Krausmann E, Cruz AM. Impact of the 11 March 2011 Great East Japan Earthquake and Tsunami on the chemical industry. Natural Hazards. 2013; 67:811–828.

[26] Adam B. The temporal gaze: The challenge for social theory in the context of GM food. British Journal of Sociology. 2000; 51(1):125–142.

[27] Oliver-Smith A, Alcántara-Ayala I, Burton I, Lavell AM. Forensic Investigations of Disasters (FORIN): A conceptual framework and guide to research IRDR FORIN Publication No 2. Beijing: Integrated Research on Disaster Risk 2016.

[28] Pollitt C. Institutional amnesia: A paradox of the 'Information Age'? Prometheus. 2000; 18(1):5–16.

[29] Quarantelli EL. Just as a disaster is not simply a big accident so a catastrophe is not just a bigger disaster. The Journal of the American Society of Professional Emergency Planner. 1996; 3:68–71.

[30] Cabinet Office Japan. White paper on traffic safety in Japan. Government of Japan 2016 http://www8.cao.go.jp/koutu/taisaku/h28kou_haku/english/wp2016-pdf.html

[31] Government of Japan. National report of Japan on disaster reduction for the World Conference on Disaster Reduction. 2005 https://www.unisdr.org/2005/mdgs-drr/national-reports/Japan-report.pdf

[32] EMDAT. The OFDA/CRED international disaster database University Catholic Louvain-Brussels Belgium http://www.emdat.be (accessed 19 January 2018)

[33] Meteorological Agency (JMA). Climate change monitoring report. 2003 through 2016. http://www.jma.go.jp/jma/en/NMHS/indexe_ccmr.html

[34] Fire Service Information Center. Fire prevention administration. Tokyo: Fire Equipment and Safety Center of Japan 2013.

[35] Kitamoto Laboratory. Japanese National Institute of Informatics. http://agora.ex.nii.ac.jp/digital-typhoon/index.html.en

[36] US Naval Weather Service/Air Weather Service Joint Typhoon Warning Center. Annual Typhoon Report http://www.usno.navy.mil/JTWC/annual-tropical-cyclone-reports

[37] Takahashi W. Divided fates of victims after the Fukushima nuclear power plant accident In: Kaneko Y, Matsuoka K, Toyoda T., editors. Asian law in disasters: Toward a human-centered recovery. New York: Routledge 2016; pp. 213–222.

[38] Changnon SA, Kunkel KE, Reinke BC. Impacts and responses to the 1995 Heat Wave: A call to action. Bulletin of the American Meteorological Society. 1996; 77(7):1497–1506.

[39] Pascal M, Wagner V, Le Tertre A, Laaidi K, Honoré C, Bénichou F, Beaudeau P. Definition of temperature thresholds: the example of the French heat wave warning system. International Journal of Biometeorology. 2013; 57:21–29. 10.1007/s00484-012-0530-1 22361805

[40] Palecki MA, Changnon SA, Kunkel KE. The nature and impacts of the July 1999 Heat Wave in the Midwestern United States: Learning from the lessons of 1995. Bulletin of the American Meteorological Society. 2001; 82:1353–1367.

[41] Nakano M, Matsueda M, Sugi M. Future projections of heat waves around Japan simulated by CMIP3 and high-resolution Meteorological Research Institute atmospheric climate models. Journal of Geophysical Research: Atmospheres. 2013; 118:3097–3109.

[42] Office of the Prime Minister of Japan Prime Ministers in history. https://japan.kantei.go.jp/cabinet/0001-30_e.html (accessed 21 May 2018)

[43] Triami Media BV Historic inflation Japan—CPI inflation. http://www.inflation.eu/inflation-rates/japan/historic-inflation/cpi-inflation-japanaspx (accessed 21 May 2018)

[44] Hood CP. Dealing with disaster in Japan: Responses to the Flight JL123 Crash. Oxon: Routledge 2012.

[45] Nohrstedt D. External shocks and policy change: Three Mile Island and Swedish nuclear energy policy. Journal of European Public Policy. 2005; 12(6):1041–1059.

[46] Reed SR. Environmental politics: Some reflections based on the Japanese case. Comparative Politics. 1981; 13(3):253–270.

[47] Grimes WW. Japan’s fiscal challenge: The political economy of reform In: Youngshik B, Pempel TJ, editors. Japan in crisis. New York: Palgrave Macmillan 2012; pp. 81–103.

[48] JGHI Japanese Government Headquarters for IDNDR. Country Report Japan. 1998. https://www.preventionweb.net/files/32471_endidndrassessmentjapan.pdf

[49] Lechat MF. The International Decade for Natural Disaster Reduction: background and objectives. Disasters. 1990; 14:1–6. 10.1111/j.1467-7717.1990.tb00967.x 20958689

[50] Hathaway OA. Path dependence in the law: The course and pattern of change in a common law legal system. Faculty Scholarship Series Paper 840. 2001. http://digitalcommons.law.yale.edu/fss_papers/840

[51] McKean MA. Environmental protest and citizen politics in Japan. Berkeley: University of California Press 1981.

[52] Hughes CW. Why Japan could revise its constitution and what it would mean for Japanese security policy. Orbis. 2006; 50(4):725–744.

[53] Ito T. Japanization is it spreading to the rest of the world? Economic Stagnation in Japan In: Cho DT, Ito T, Mason A, editors. Exploring the causes and remedies of Japanization. Cheltenham: Edward Elgar 2018; pp. 17–55.

[54] Grimes WW. Internationalization of the Yen and the new politics of monetary insulation In: Kirshner J., editor. Monetary orders: Ambiguous economics ubiquitous politics. Ithaca: Cornell University Press 2003 pp. 172–194.

[55] Calder KE. Crisis and compensation: Public policy and political stability in Japan. Princeton: Princeton University Press 1988.

[56] Scheuerman W. Liberal democracy and the social acceleration of time. Baltimore: The Johns Hopkins University Press 2004.

[57] Kilian L. A comparison of the effects of exogenous oil supply shocks on output and inflation in the G7 countries. Journal of the European Economic Association. 2008; 6:78–121.

[58] Lewis J, Kelman I. The good, the bad and the ugly: Disaster Risk Reduction (DRR) versus Disaster Risk Creation (DRC). PLOS Currents Disasters. 2012 6 21 Edition 1. 10.1371/4f8d4eaec6af8 22919564 PMC3423310

[59] Kelman I, Gaillard JC, Lewis J, Mercer J. Learning from the history of disaster vulnerability and resilience research and practice for climate change. Natural Hazards. 2016; 82:129–143.

[60] Becker P. Grasping the hydra: The need for a holistic and systematic approach to disaster risk reduction. Jàmbá: Journal of Disaster Risk Studies. 2009; 2(1). https://journals.co.za/content/jemba/2/1/EJC51158

